# Analysis and visualization of sleep stages based on deep neural networks

**DOI:** 10.1016/j.nbscr.2021.100064

**Published:** 2021-03-12

**Authors:** Patrick Krauss, Claus Metzner, Nidhi Joshi, Holger Schulze, Maximilian Traxdorf, Andreas Maier, Achim Schilling

**Affiliations:** a Neuroscience Lab, Experimental Otolaryngology, University Hospital Erlangen, Germany; b Cognitive Computational Neuroscience Group at the Chair of English Philology and Linguistics, Friedrich-Alexander University Erlangen-Nürnberg (FAU), Germany; c Cognitive Neuroscience Center, University of Groningen, the Netherlands; d Biophysics, Friedrich-Alexander University Erlangen-Nürnberg (FAU), Germany; e Department of Otolaryngology, Head and Neck Surgery, University Hospital Erlangen, Germany; f Machine Intelligence, Friedrich-Alexander University Erlangen-Nürnberg (FAU), Germany; g Laboratory of Sensory and Cognitive Neuroscience, Aix-Marseille University, Marseille, France

**Keywords:** Sleep stage scoring, Hypnodensity graphs, Multidimensional scaling (MDS), Electroencephalography (EEG), Artificial neural networks, Deep learning, Polysomnography (PSG), Sleep cycle analysis

## Abstract

Automatic sleep stage scoring based on deep neural networks has come into focus of sleep researchers and physicians, as a reliable method able to objectively classify sleep stages would save human resources and simplify clinical routines. Due to novel open-source software libraries for machine learning, in combination with enormous recent progress in hardware development, a paradigm shift in the field of sleep research towards automatic diagnostics might be imminent. We argue that modern machine learning techniques are not just a tool to perform automatic sleep stage classification, but are also a creative approach to find hidden properties of sleep physiology. We have already developed and established algorithms to visualize and cluster EEG data, facilitating first assessments on sleep health in terms of sleep-apnea and consequently reduced daytime vigilance. In the following study, we further analyze cortical activity during sleep by determining the probabilities of momentary sleep stages, represented as hypnodensity graphs and then computing vectorial cross-correlations of different EEG channels. We can show that this measure serves to estimate the period length of sleep cycles and thus can help to find disturbances due to pathological conditions.

## Highlights

• Artificial intelligence leads to a paradigm shift in sleep research.
• Sleep stage scoring with deep neural networks is robust and reliable.
• Neural networks find embeddings of EEG raw data.
• Probabilities of momentary sleep stages can be visualized as hypnodensity graphs.
• Cross-correlations of sleep stage probability vectors provide an estimate of sleep cycle period length.

## 1 Introduction

Sleep stage scoring is a standard procedure and part of every polysomnographic analysis (Bradley and Peterson, 2008; Burns et al., 2008). Up to now, sleep stage scoring based on physiological signals (EEG: electroencephalography, EMG: electromyography, EOG: electroocculugraphy) is performed by experienced clinicians, which do the classification by hand according to the AASM guidelines (Berry et al., 2012). However, this procedure is time consuming and highly prone to errors resulting in high inter-rater variability (Danker-Hopfe et al., 2004, 2009; Wendt et al., 2015). To overcome these limitations, machine learning algorithms were applied. These algorithms are still not fully trusted by clinicians as they act as a black-box, from which no one knows what the internal criteria for the different sleep stages really are. Thus, one core-problem of modern machine learning research has met clinical routines, the so called black-box problem (KrauseAdam and Ng, 2016) which in other scientific fields is also called the opacity debate (De Laat, 2018). This problem can be tackled by simply using hand-crafted features such as time tags of K-complexes or sleep spindles as neural network input (Phan et al., 2018; Boostani et al., 2017), (for an example see (PhanQuan et al., 2013)). However, this procedure is very elaborate and the results are still not satisfying. In times of rising computing power and increasing storage capacities, a Big Data approach where neural networks are finding the best features in a self-organized way seems to be more promising. In order to analyze these features and the emerging internal representations, novel concepts for data visualization are needed. A sophisticated dimensionality reduction method for visualising high dimensional data is a demanding task, as certain popular methods can lead to pseudo clustering of data points caused by the initial conditions and the used hyper-parameters of the projection algorithm. A famous example is t-distributed stochastic neighbor embedding (t-SNE (van der Maaten and Hinton, 2008),), which is highly unstable and extremely dependent on the initialization and choice of parameters (Martin et al., 2016).

In previous studies, we developed several approaches to statistically analyze and visualize high-dimensional neural data (Krauss et al., 2018a; Schilling et al., 2018). We developed a statistical method for analyzing and comparing high-dimensional spatio-temporal cortical activation patterns for different auditory and somatosensory stimulus conditions in rodents and humans (Krauss et al., 2018a). The cortical activity patters were represented by amplitude vectors calculated via a sliding window method (for the exact procedure see (Krauss et al., 2018a)). We could already demonstrate that this method can discriminate different sleep stages in human EEG recordings (Krauss et al., 2018b). Furthermore, we could analyze the microstructure of cortical activity during sleep and found that it reflects respiratory events and the state of daytime vigilance (Traxdorf et al., 2019). Recently, our method has been generalized, and can now be used to analyze and compare representations of artificial neural networks (Schilling et al., 2018). In the following study, we first illustrate how to visualize the representations of EEG data gained from different layers of artificial neural networks (sleep stage embeddings). Furthermore, we demonstrate that these complex representations cluster better in higher layers of the artificial neural networks, quantified by the generalized discrimination value (GDV, see also (Schilling et al., 2018)). Subsequently, we visualize the output of the last layer of the neural network, and thus the momentary probabilities of the predicted sleep stages, as so called hypnodensity graphs (as introduced by (Jens et al., 2018)). Finally, we use these probability vectors to calculate vectorial cross-correlations, in order to analyze the period length of sleep cycles.

## 2 Methods

### 2.1 Data base

The study was conducted in the Department of Otorhinolaryngology, Head Neck Surgery, of the Friedrich-Alexander University Erlangen-Nürnberg (FAU), following approval by the local Ethics Committee (323–16 Bc). All 68 participating subjects, 46 male and 22 female, mean age 32.5±11.5 years, were recruited by the Department of Otorhinolaryngology, Head and Neck Surgery. Written informed consent was obtained from the participants before the cardiorespiratory polysomnography (PSG). Inclusion criterion for this study was age between 21 and 80 years. Exclusion criteria were a positive history of misuse of sedatives, alcohol or addictive drugs and untreated sleep disorders. Data analysis was carried out during time in bed of the subjects, accumulating to a total recording time of approximately 510 h.

Each of the 68 data sets comprising a full night of sleep consisted of 3 channels of EEG recordings (F4-M1, C4-M1, O2-M1) and the corresponding sleep stages. Sleep stages were analyzed and scored visually in 30 s epochs according to the AASM criteria (Version 2.1, 2014) by a sleep specialist accredited by the German Sleep Society (DGSM) (ConradIber, 2007; Berry et al., 2012). Thereby, typical artifacts (Tatum et al., 2011) have been marked and removed subsequently for further analysis and processing steps.

### 2.2 Sleep stage classification with neural networks

A deep neural network was trained on single channel sleep EEG data. Therefore, we used the total number of 68 data sets and split them randomly in 54 training data sets (80%) and 14 test data sets (20%), as this is the standard approach in machine learning and pattern recognition (Bishop, 2006; Goodfellow et al., 2016). For each data set, the three different EEG channels were concatenated, yielding a corresponding single channel data set with three times the duration of the original data set. As labels for supervised training and as ground truth for the test data set we used the sleep stages from visual analysis and scoring.

**Fig. 1:**
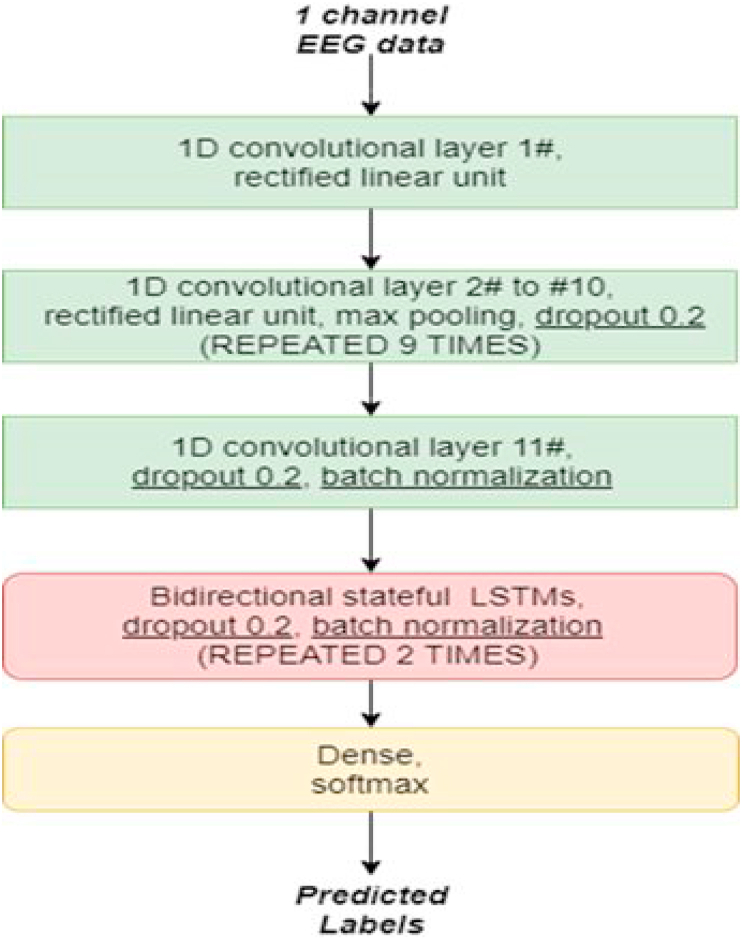
**Network architecture.** Building blocks of the deep neural network trained on sleep stage classification of EEG data. The network consists of eleven 1D convolutional layers, nine max pooling layers, 2 layers of bidirectional stateful LSTMs, and a fully connected classification layer with softmax output.

The network consisting of several convolutional layers (LeCun Bengioet al., 1995) and two bidirectional stateful LSTM layers (Hochreiter and Schmidhuber, 1997) was trained with error back propagation to classify the sleep stages (for exact network architecture see Fig. 1). The convolutional layers allow to extract relevant features for classification in an unsupervised way (LeCun et al., 2015), i.e. without the need for manually extracting features like frequency power spectra or amplitudes. Recurrent network layers like the here used LSTM layers allow to extract and capture temporal information [] from the sequences of sleep stages.

### 2.3 Software resources

The software is written using the programming language python 3.6, together with the KERAS AI library (Chollet, 2018) and tensorFlow backend. The data is pre-processed using Numpy (Stéfan van der Walt and Varoquaux, 2011). For multi-dimension-scaling (MDS) the scikit-learn library (Pedregosa et al., 2011) is used and the visualization of the results is done using the matplotlib library (Hunter, 2007).

### 2.4 Hypnodensity correlations

The hypnodensity cross-correlation for lag-time τ is defined as

(1) $$r_{x,y}\left(\tau \right)=\frac{\frac{1}{T-\tau }\sum_{t=1}^{T-\tau }\left(x_{t}-\bar{x}\right)\circ \left(y_{t+\tau }-\bar{y}\right)}{\sqrt{\frac{1}{T}\sum_{t=1}^{T}||x_{t}-\bar{x}|{|}^{2}}\cdot \sqrt{\frac{1}{T}\sum_{t=1}^{T}||y_{t}-\bar{y}|{|}^{2}}}$$

and the hypnodensity auto-correlation for lag-time τ is defined as

(2) $$r_{x,x}\left(\tau \right)=\frac{\frac{1}{T-\tau }\sum_{t=1}^{T-\tau }\left(x_{t}-\bar{x}\right)\circ \left(x_{t+\tau }-\bar{x}\right)}{\frac{1}{T}\sum_{t=1}^{T}||x_{t}-\bar{x}|{|}^{2}}$$

with $x_{t}={\left(x_{1},x_{2},x_{3},x_{4},x_{5}\right)}_{t}^{T}$ and $y_{t}={\left(y_{1},y_{2},y_{3},y_{4},y_{5}\right)}_{t}^{T}$ being 5-dimensional vectors that contain the momentary probabilities of all sleep stages (indices 1 to 5) at time *t* for EEG channels *x* and *y*, respectively, and the mean probability vectors $\bar{x}=\frac{1}{T}\sum_{t=1}^{T}x_{t}$ and $\bar{y}=\frac{1}{T}\sum_{t=1}^{T}y_{t}$. Here, ∘ is the dot product, and |||| the vector norm.

## 3 Results

### 3.1 Sleep stage embeddings

We could show that even a single EEG channel contains enough information to classify different sleep stages. We used several convolutional blocks, which transform the input EEG data into high-dimensional complex features. A procedure referred to as *feature space embedding*. Thus, in contrast to handcrafted features we make the neural network to find its own features. This higher order features lead to a good separability of the transformed EEG vectors (neural network layer output or representation) belonging to different sleep stages. In the following, we refer to these self-organized representations as *sleep stage embeddings*.

**Fig. 2:**
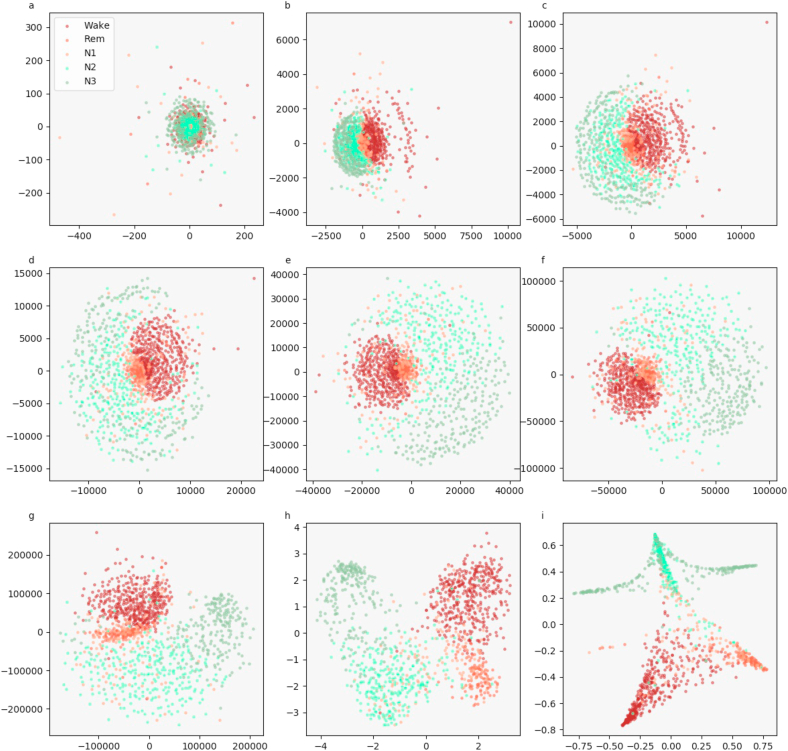
**Sleep stage embeddings.** MDS visualization of EEG data representations (embeddings) within the hidden layers of the deep neural network: input layer (a), max pooling layers (b–g), last LSTM layer (h), and softmax layer (i). The hidden layers (b–g) lead to increasingly better separability of sleep stages compared to z-scored raw EEG data (a). The softmax classification layer (i) normalizes the data to a value range of [0,1]. Note that absolute coordinates of points in MDS projections have no particular meaning other than scaling relative distances between any pair of points.

**Fig. 3:**
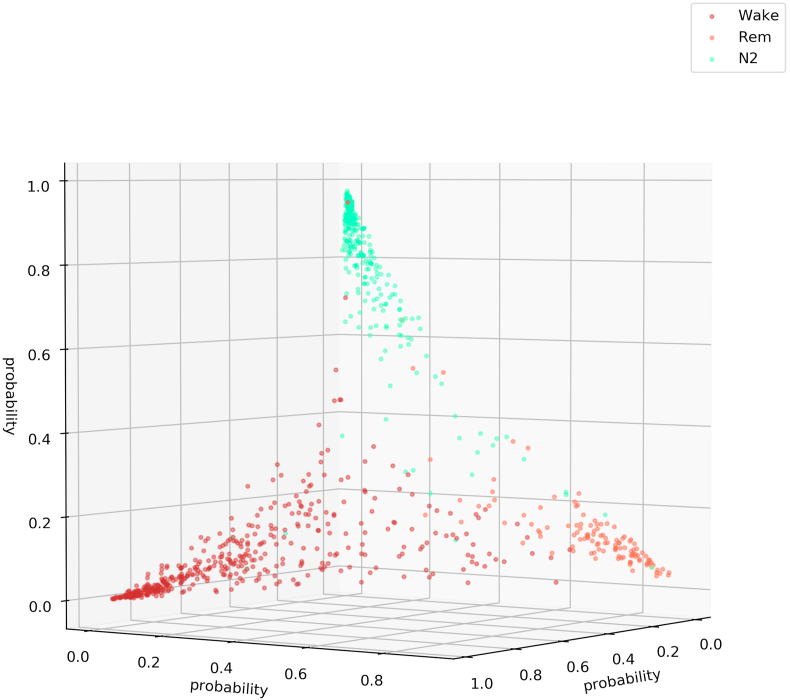
**Softmax output for three sleep stages (Wake, REM, N2).** This figure illustrates that the softmax output spans a hyper-plane (in 3D a 2D hyper-plane with 3 corners). Note that the possible outputs lie in the volume below the hyper-plane. Not all points lie on the plane as confusions with sleep stage N3 or N1 cannot be shown in a 3D plot. Thus, if the softmax output would for example point to sleep stage N3 the point would lie near the origin (0,0,0). Note that absolute coordinates of points in MDS projections have no particular meaning other than scaling relative distances between any pair of points.

**Fig. 4:**
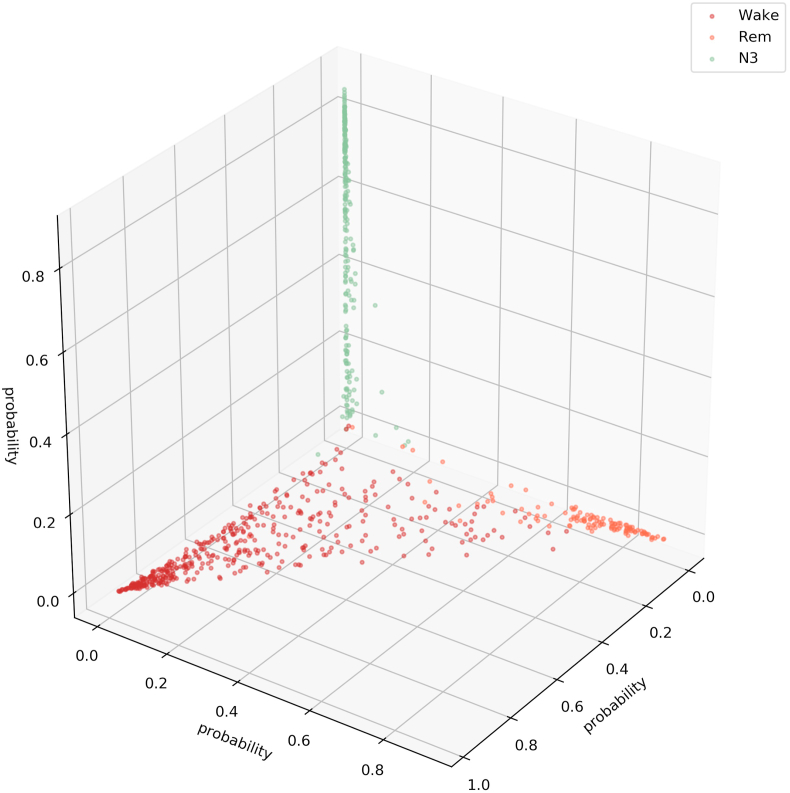
**Softmax output of for three sleep stages (Wake, REM, N3).** This figure illustrates that the softmax output spans a hyper-plane (in 3D a 2D hyper-plane with 3 corners). It can be seen that the sleep stage N3 is not confused with the Wake or the REM state. Note that absolute coordinates of points in MDS projections have no particular meaning other than scaling relative distances between any pair of points.

We visualize the sleep stage embeddings by a dimensionality reduction into 2D, using multidimensional scaling (MDS) (Krauss et al., 2018a) (Fig. 2). Moreover, we evaluate the generalized discrimination value (GDV), which quantifies separability of data classes in high-dimensional state spaces (Schilling et al., 2018). The MDS plots show that the convolutional layers lead to better separability (Fig. 2b–g) compared to z-scored raw EEG data (network input, Fig. 2a). Furthermore, the LSTM layer transforms the data so that the different classes are linearly separable (Fig. 2h). The softmax classification layer normalizes the data (value range [0,1]). The shape of the clusters in Fig. 2i can be explained by the fact that the softmax layers places the different classes at the 5 edges of a 4D-hyper-plane (example for 3D projections shown in Fig. 3, Fig. 4). As the probabilities of all 5 sleep stages have to sum up to a value of 1, the range of possible softmax outputs is located on this hyper-plane. The MDS projection of the hyper-plane in 2D leads to the shown patterns (Fig. 2i). Note that, Fig. 2, Fig. 3, Fig. 4) were generated using one sample training data set. Labels are neither used during embedding in the neural network layers, nor for projection, but they serve only for color coding the data points after embedding and projection.

**Fig. 5:**
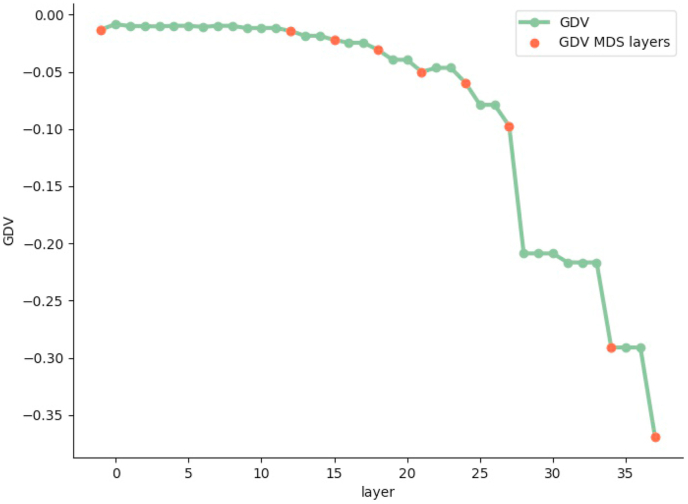
**Separability of sleep stages.** Separability increases with increasing layer depth. Note that, a GDV of 0 corresponds to non-separable data classes, whereas a GDV of −1 corresponds to a perfect data class separation. Red dots refer to the layer outputs shown in 2 (first red dot: input, 2nd-7th: max pooling layers, 8th: 2nd LSTM layer, 9th: softmax output layer). (For interpretation of the references to color in this figure legend, the reader is referred to the Web version of this article.)

The quantification of the class separability via the GDV (Fig. 2, red dots: input, 6 max-pooling layers, the second LSTM layer, the softmax output layer), shows that the convolutional layers perform data pre-processing. In a previous study, we could demonstrate that the GDV has to overcome an “energy barrier”, when the data structure is complex. Thus, the number of layers where the GDV only slightly decreases depends on data complexity. As complex features are needed to classify the data, there are relatively many initial layers with small GDV decrease (cf. Fig. 5). The LSMT layer leads to a clear increase of separability, i.e. drop in the GDV value (8th red point in Fig. 5, compare also Fig. 2 i). The softmax layer causes a further decrease of the GDV value.

### 3.2 Hypnodensity graphs

An efficient illustration of the softmax layer output are so called hypnodensity graphs (Jens et al., 2018). Here, the probabilities for all sleep stages at each time point are shown instead of only the most probable sleep stage, as it is done in traditional hypnograms. This is an elegant approach, since the probabilities (output of softmax layer) can be compared with the uncertainties, i.e. different assessments of different somnologists, which cause the relatively high inter-rater-variability (Danker-Hopfe et al., 2004, 2009; Wendt et al., 2015).

**Fig. 6:**
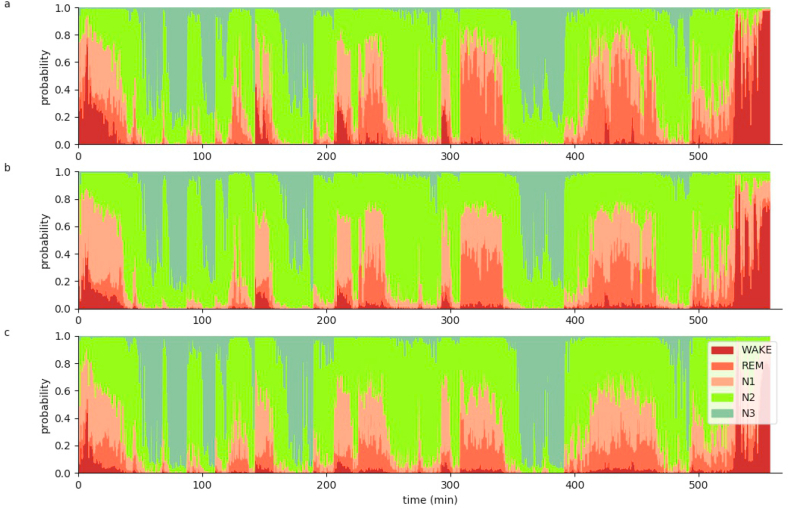
**Hypnodensity graph.** Hypnodensity graph of subject 55 with a temporal resolution of 5 s separately evaluated for the three different EEG channels C4 (a), F4 (b) and O2 (c). The probabilities for all sleep stages at each time point are shown. Each EEG channel is sufficient to perform sleep stage classification, as the hypnodensity graphs are similar.

Additionally, the automatic sleep stage classification can be used to evaluate the data with higher temporal resolution. Even though, the network has been trained on labeled data with a temporal resolution of 30 s which is in accordance with AASM scoring rules (Berry et al., 2012), we evaluate sleep stage probabilities predicted by the neural network with a temporal resolution of 5 s, using a sliding window approach (Fig. 6 and Fig. S1 – S10). Thus, we still analyzed 30 s windows, yet with 5 s steps. The predicted sleep stage probabilities were assigned to the respective center of each window of 30 s width.

The hypnodensity plots show that each EEG channel is sufficient to perform sleep stage classification, as the hypnodensity graphs are very similar (Fig. 6) for all the channels. Interestingly, the sleep stage *wake* seems to be slightly over-represented in channel C4 (6a), whereas in channel O2, *N3* seems to slightly dominate (6c). The similarity between channels can be quantified, for example using pairwise cross-correlations.

### 3.3 Hypnodensity based sleep cycle period length analysis

On the basis of these EEG channel specific hypnodensity graphs, sleep stage probabilities across and within channels can be compared (see Methods). For this purpose we calculate generalized vector auto- and cross-correlations of the 5-dimensional probability vectors for the 3 EEG channels of each data set. This correlations provide valuable information on neuronal activity patterns during sleep. In particular, all auto-as well as cross-correlations have a global maximum, which is near to the value 1, at zero lag time. This means that there is no time shift of the neuronal patterns between the different channels. Furthermore, the high correlation coefficients at lag time zero indicate that all channels are sufficient to perform sleep scoring, which could already been predicted by the hypnodensity graphs. Local side-maxima indicate repetitions of neuronal patterns as they occur in repeating sleep-cycles.

**Fig. 7:**
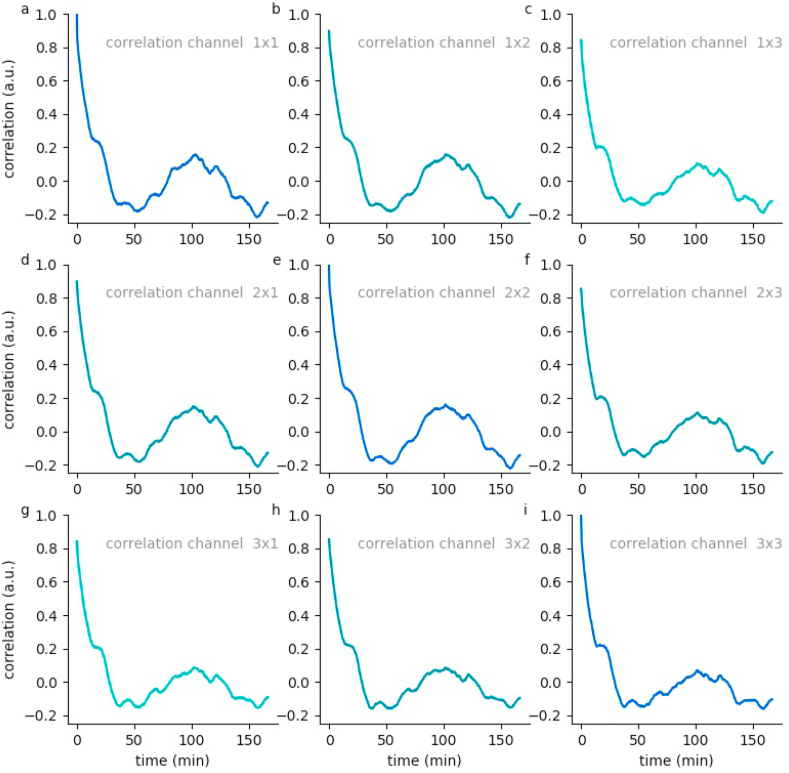
**Hypnodensity cycles.** Temporal auto- and cross correlations of 5-dimensional hypnodensity probability vectors of sleep stages. In subject 55, a local maximum at a lag-time of about 100 min can be observed, indicating an individual period length of sleep cycles of 100 min.

**Fig. 8:**
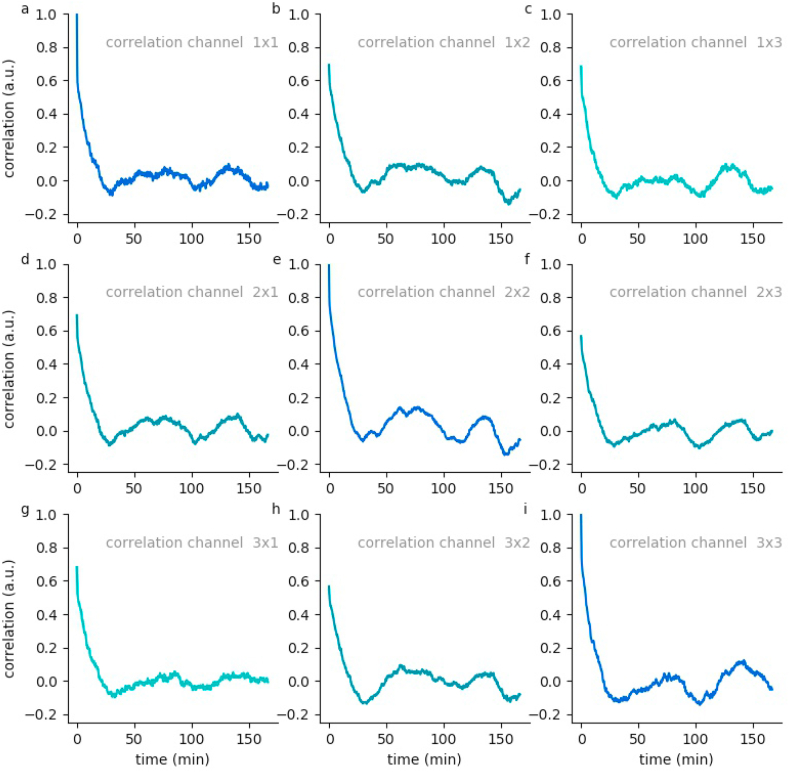
**Hypnodensity cycles.** Temporal auto- and cross correlations of 5-dimensional hypnodensity probability vectors of sleep stages. In subject 59, two local maxima at lag-times of about 75 and 150 min can be observed, indicating an individual period length of sleep cycles of 75 min.

For subject 55, the auto and cross-correlations show a local maximum at a lag-time of about 100 min (Fig. 7). This means that after this period of time the sleep stages repeat. In contrast, data of subject 59 show two local maxima at lag-times of 75 and 150 min, respectively (Fig. 8). Hence, this subject's sleep stage period length is about 75 min. These estimated period lengths have been confirmed by the sleep stage scoring based on visual analysis performed by sleep specialists. This means that our novel method helps to reliably estimate the individual period length of sleep cycles. Further data are shown in Figs. S11 – S19.

## 4 Discussion

In this study, we present novel approaches for the evaluation and visualization of neural data recorded during human sleep. In contrast to numerous previous studies and network architectures used to perform automatic sleep stage scoring (e.g. (Ebrahimi et al., 2008; Koley and Dey, 2012; Zhang and Wu, 2017; Amiya et al., 2018; Alickovic and Subasi, 2018; Zhang et al., 2016)), we provide approaches helping to interpret and understand the network output.

Thus, we illustrate how deep neuronal networks automatically find features that help to separate different sleep stages based on single channel EEG data. That this representations actually lead to a good separability of the different sleep stages is visualized using MDS plots and quantified using the GDV value.

The softmax output, i.e. the predicted probabilities for each sleep stage at every time step, is visualized with hypnodensity graphs (Jens et al., 2018) a sophisticated way to show the uncertainties in sleep stage classification. The calculation of the vectorial auto- and cross-correlations within and between channels, as introduced in this study, is a tool to gain more insight into the architecture of sleep, e.g. sleep cycles. We propose the auto-correlation plots as novel tool to estimate the average period length of sleep stage cycles, and to find disturbances in the period length of sleep cycles, e.g. as an indicator for pathological conditions, for example in the case of patients with obstructive sleep apnea (OSAS). Here, sleep is dominated by events like arousals and short periods of wakefulness. These effects “destroy” the ordered sequence of sleep stages of normal sleep cycles. The cross-correlation plots between different recording channels may serve to shed light into phenomena like local or fragmented sleep. The global maximum of cross-correlation plots, especially if the corresponding lag-time is different from zero, indicates travelling waves across the cerebral cortex, or the order at which the different regions change sleep stages.

Another interesting extension of the correlation method for future studies would be the application to non-stationary cases, i.e. changing period lengths over night. For instance, calculating the correlation only for subsequent parts of the entire recording time would yield different lag-times at maximum correlation, i.e. period length estimates, for each different part. If correlation analysis is initially performed over the complete night, as shown above, this yields the average cycle time, and hence gives a hint on how to choose the duration of the different parts to be analyzed. Alternatively, a sliding window approach could be applied, yielding a time-dependent period length, i.e. lag-time at maximum correlation. Even more sophisticated would be the application of super-statistical methods (Metzner et al., 2015), which are designed to detect the temporal change of statistical parameters in complex systems.

This study is in line with numerous previous studies trying to use artificial neural networks as a model (KriegeskortePamela, 2018; Schilling et al., 2020a; Gerum et al., 2020; Krauss et al., 2019a, 2019b; Krauss and Maier, 2020), and as a tool (Schilling et al., 2020b) to analyze and understand brain activity. This combined approach at the intersection of neuroscience and artificial intelligence is crucial to make further progress in the field, as traditional popular analysis methods have been proven to be not sufficient to understand brain dynamics (Jonas and Paul Kording, 2017; Brown, 2014; Lazebnik, 2002).

## CRediT authorship contribution statement

**Patrick Krauss:** Conceptualization, Formal analysis, Funding acquisition, Investigation, Methodology, Project administration, Software, Supervision, Visualization, Writing – original draft, Writing – review & editing. **Claus Metzner:** Formal analysis, Investigation, Methodology, Software, Validation, Writing – review & editing. **Nidhi Joshi:** Formal analysis, Software, Visualization. **Holger Schulze:** Resources. **Maximilian Traxdorf:** Data curation, Resources. **Andreas Maier:** Investigation, Resources, Supervision, Validation. **Achim Schilling:** Conceptualization, Formal analysis, Investigation, Methodology, Software, Visualization, Writing – original draft.

## Declaration of competing interest

All authors declare no competing financial interests.
