# Supplementary material for: Analysis and visualization of sleep stages based on deep neural networks
Source: Neurobiol Sleep Circadian Rhythms. 2021 Mar 12;10:100064. doi: 10.1016/j.nbscr.2021.100064 (PMC7973384; doi:10.1016/j.nbscr.2021.100064)
Supplement: Multimedia component 1 [file mmc1.pdf]

Supplements to: Analysis and Visualization of Sleep Stages based on Deep Neural Networks

Patrick Krauss[1,2,3], Claus Metzner[1,4], Nidhi Joshi[1], Holger Schulze[1], Maximilian Traxdorf[5], Andreas Maier[6], and Achim Schilling[1,2,7]

[1]Neuroscience Lab, Experimental Otolaryngology, University Hospital Erlangen, Germany
[2]Cognitive Computational Neuroscience Group at the Chair of English Philology and Linguistics, Friedrich-Alexander University Erlangen-Nürnberg (FAU), Germany
[3]Cognitive Neuroscience Center, University of Groningen, The Netherlands
[4]Chair of Biophysics, Friedrich-Alexander University Erlangen-Nürnberg (FAU), Germany
[5]Department of Otolaryngology, Head and Neck Surgery, University Hospital Erlangen, Germany
[6]Chair of Machine Intelligence, Friedrich-Alexander University Erlangen-Nürnberg (FAU), Germany
[7]Laboratory of Sensory and Cognitive Neuroscience, Aix-Marseille University, Marseille, France

March 5, 2021

Keywords:
sleep stage scoring, hypnodensity graphs, multidimensional scaling (MDS), electroencephalography (EEG), artificial neural networks, deep learning, polysomnography (PSG), sleep cycle analysis

Supplements

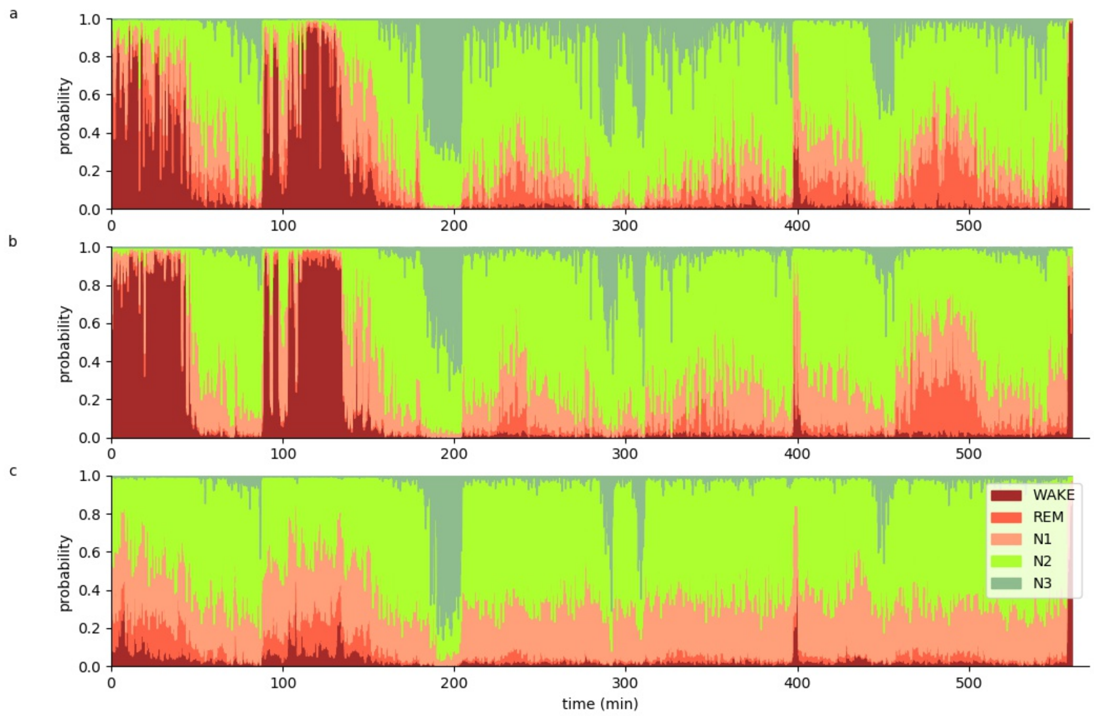

Figure S1: **Hypnodensity graph.**
Hypnodensity graph of subject 56 with a temporal resolution of 5 seconds separately evaluated for the three different EEG channels C4 (a), F4 (b) and O2 (c).

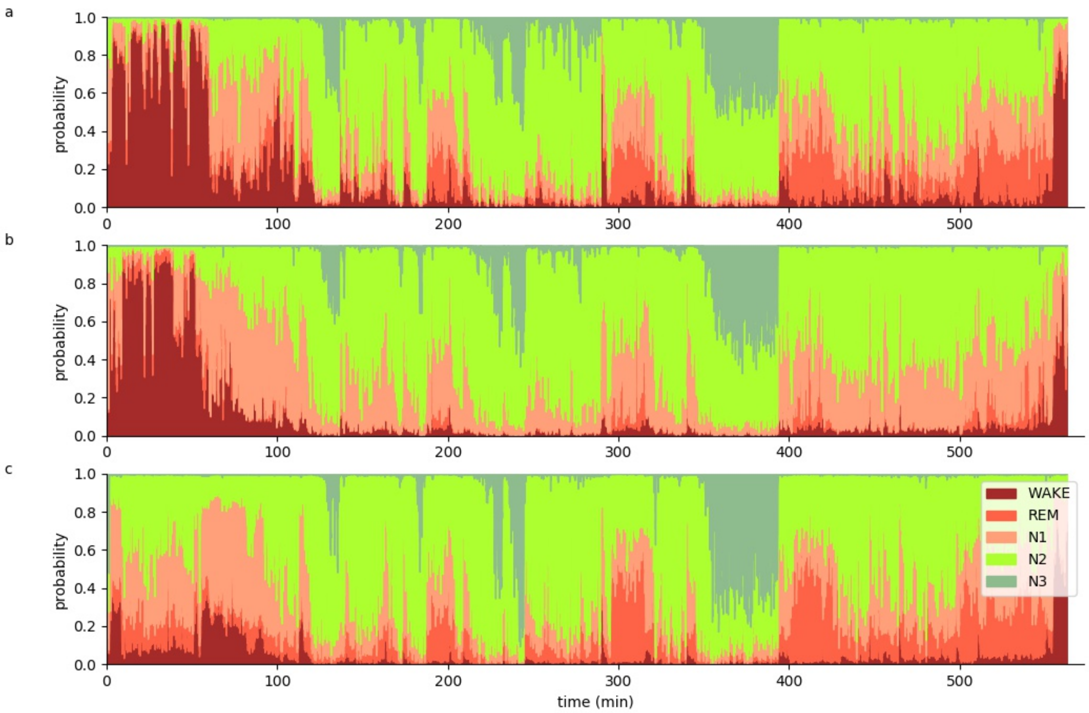

Figure S2: **Hypnodensity graph.**
Hypnodensity graph of subject 57 with a temporal resolution of 5 seconds separately evaluated for the three different EEG channels C4 (a), F4 (b) and O2 (c).

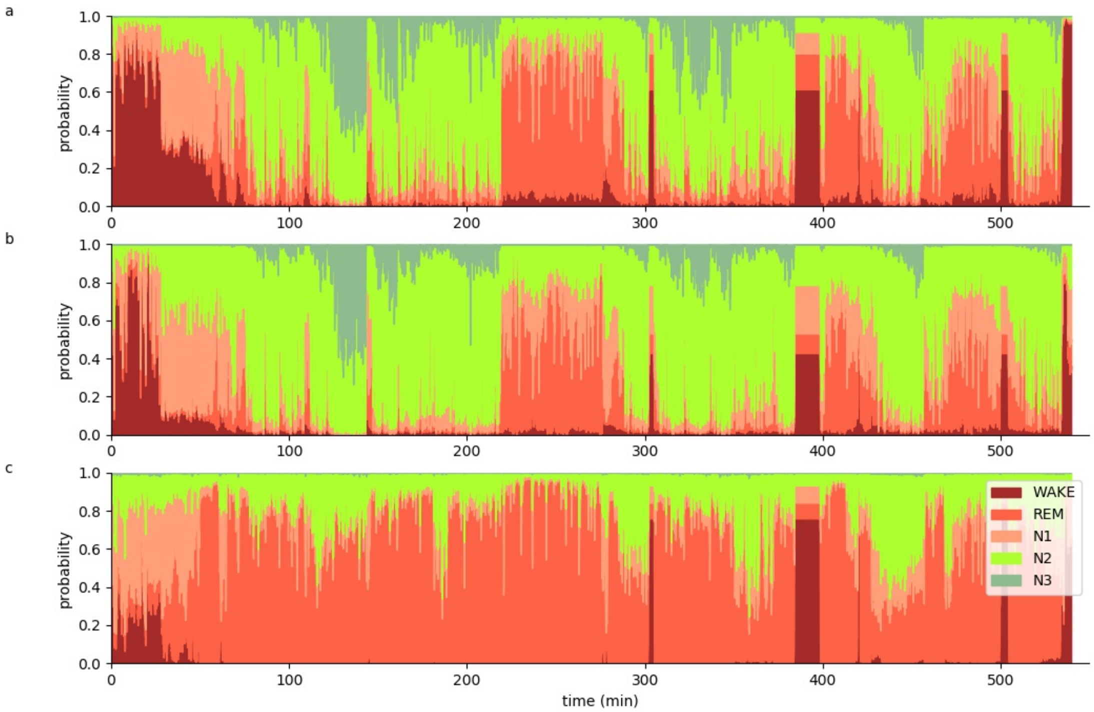

Figure S3: **Hypnodensity graph.**
Hypnodensity graph of subject 58 with a temporal resolution of 5 seconds separately evaluated for the three different EEG channels C4 (a), F4 (b) and O2 (c).

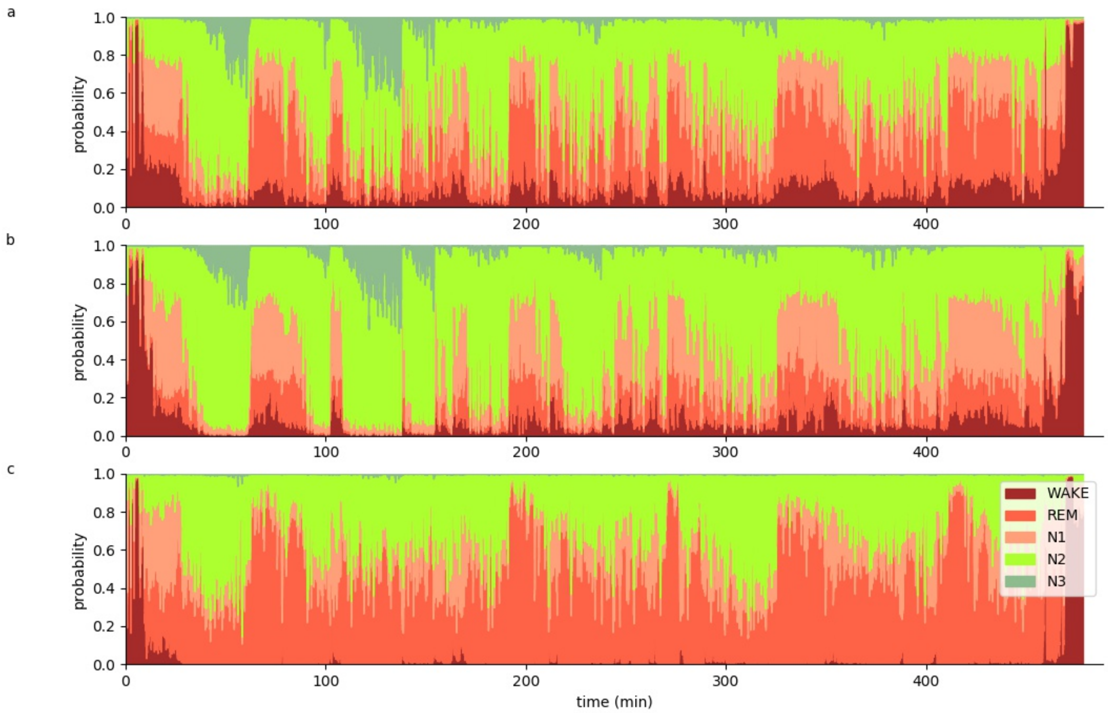

Figure S4: **Hypnodensity graph.**
Hypnodensity graph of subject 59 with a temporal resolution of 5 seconds separately evaluated for the three different EEG channels C4 (a), F4 (b) and O2 (c).

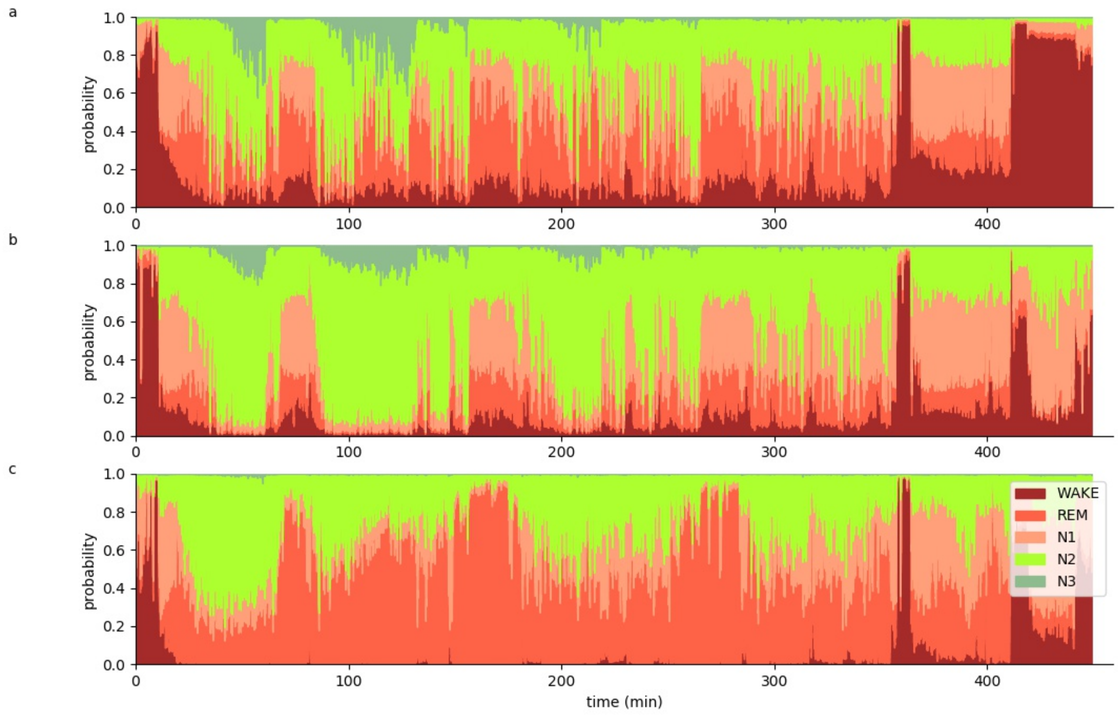

Figure S5: **Hypnodensity graph.**
Hypnodensity graph of subject 60 with a temporal resolution of 5 seconds separately evaluated for the three different EEG channels C4 (a), F4 (b) and O2 (c).

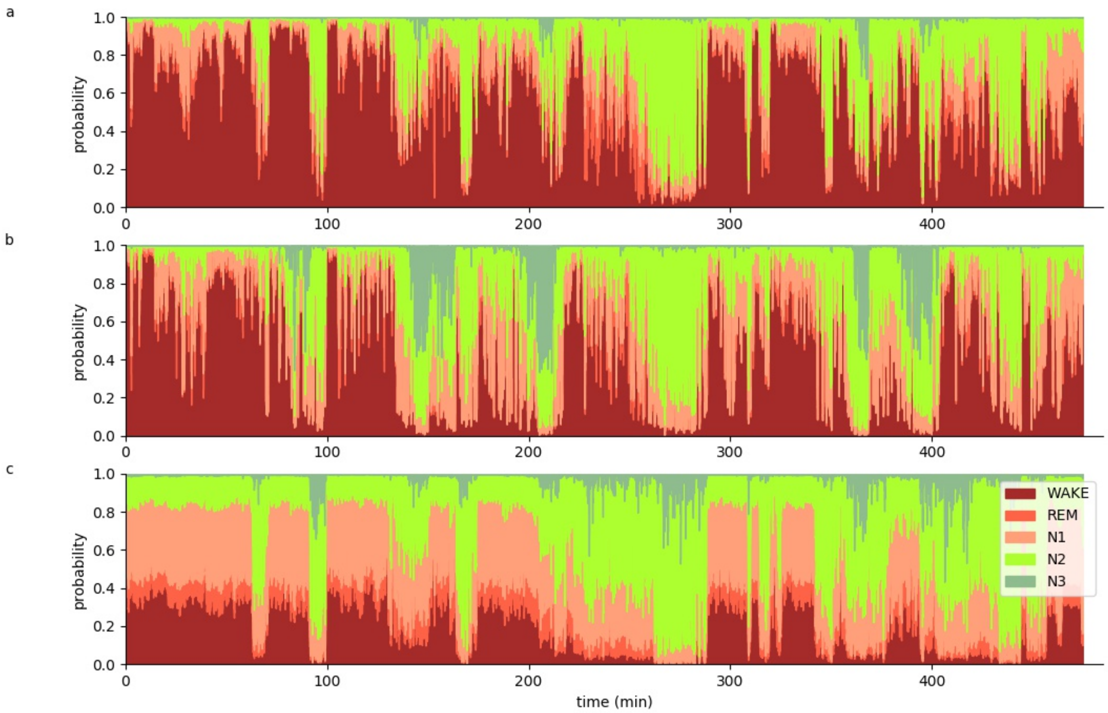

Figure S6: **Hypnodensity graph.**
Hypnodensity graph of subject 61 with a temporal resolution of 5 seconds separately evaluated for the three different EEG channels C4 (a), F4 (b) and O2 (c).

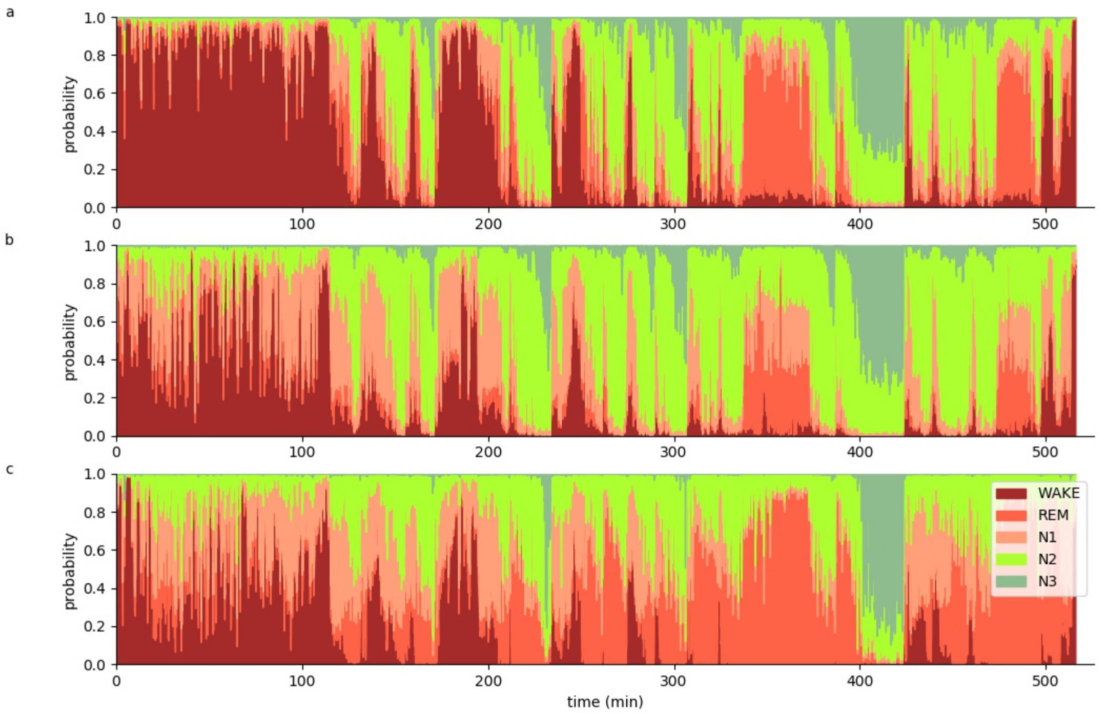

Figure S7: **Hypnodensity graph.**
Hypnodensity graph of subject 62 with a temporal resolution of 5 seconds separately evaluated for the three different EEG channels C4 (a), F4 (b) and O2 (c).

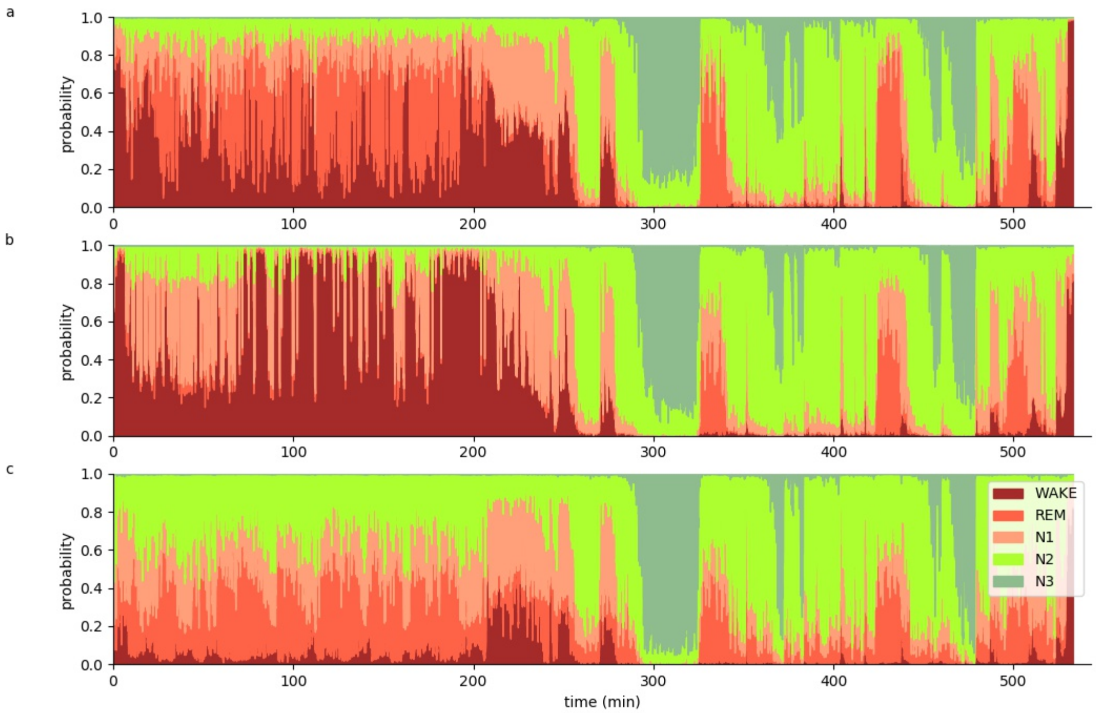

Figure S8: **Hypnodensity graph.**
Hypnodensity graph of subject 63 with a temporal resolution of 5 seconds separately evaluated for the three different EEG channels C4 (a), F4 (b) and O2 (c).

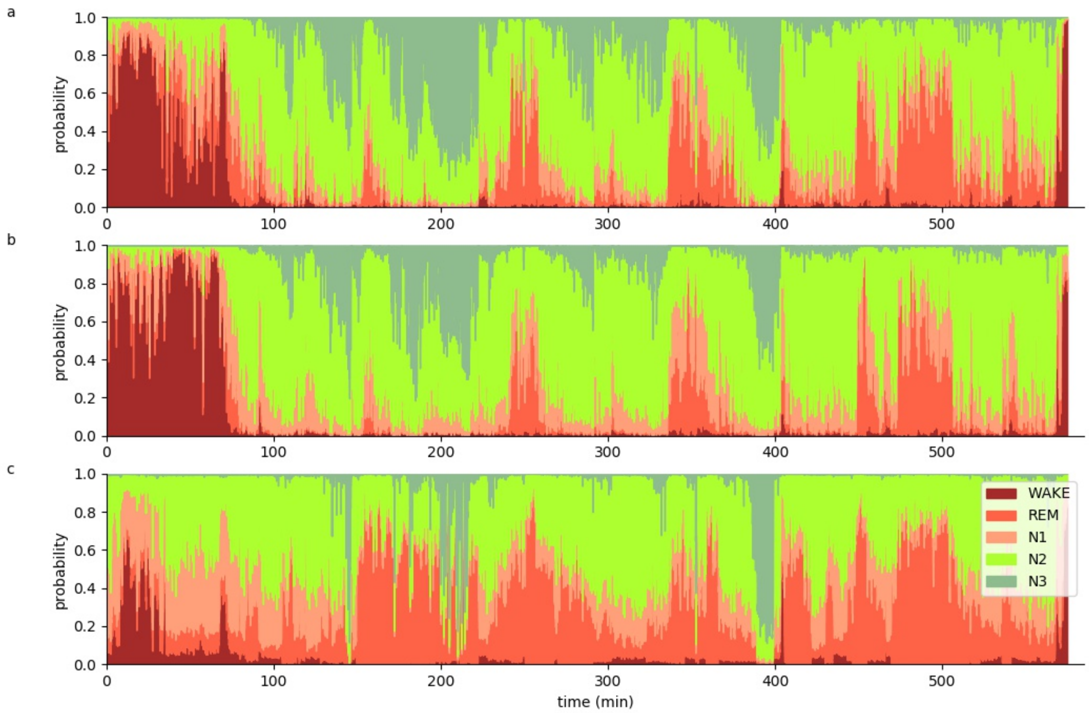

Figure S9: **Hypnodensity graph.**
Hypnodensity graph of subject 64 with a temporal resolution of 5 seconds separately evaluated for the three different EEG channels C4 (a), F4 (b) and O2 (c).

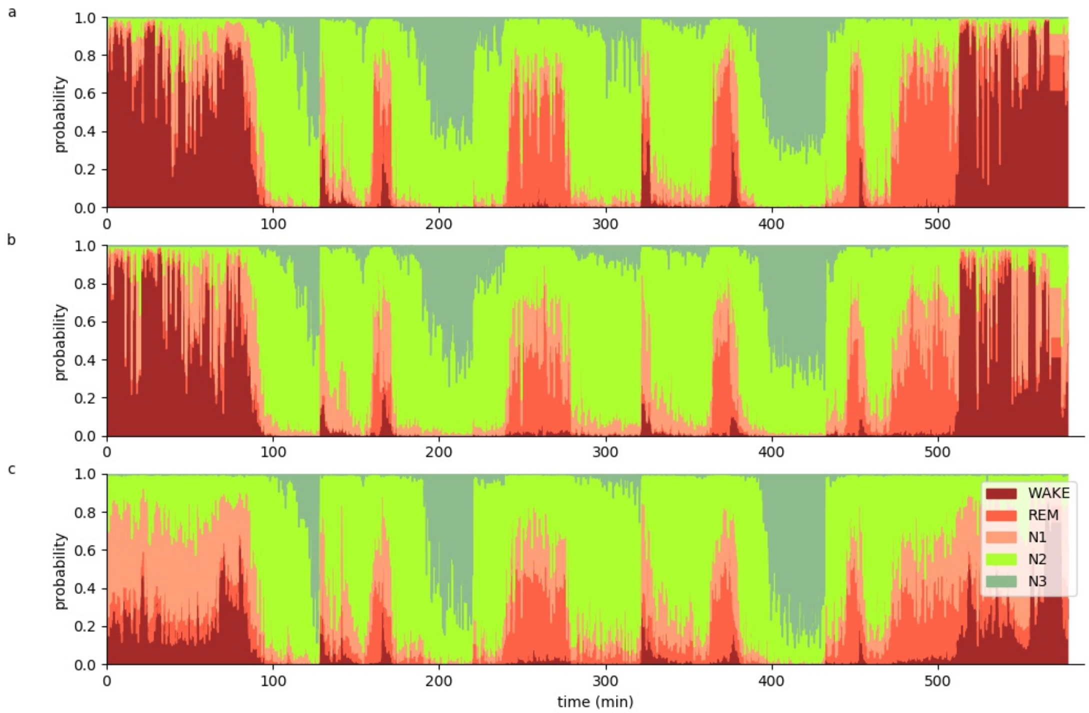

Figure S10: **Hypnodensity graph.**
Hypnodensity graph of subject 65 with a temporal resolution of 5 seconds separately evaluated for the three different EEG channels C4 (a), F4 (b) and O2 (c).

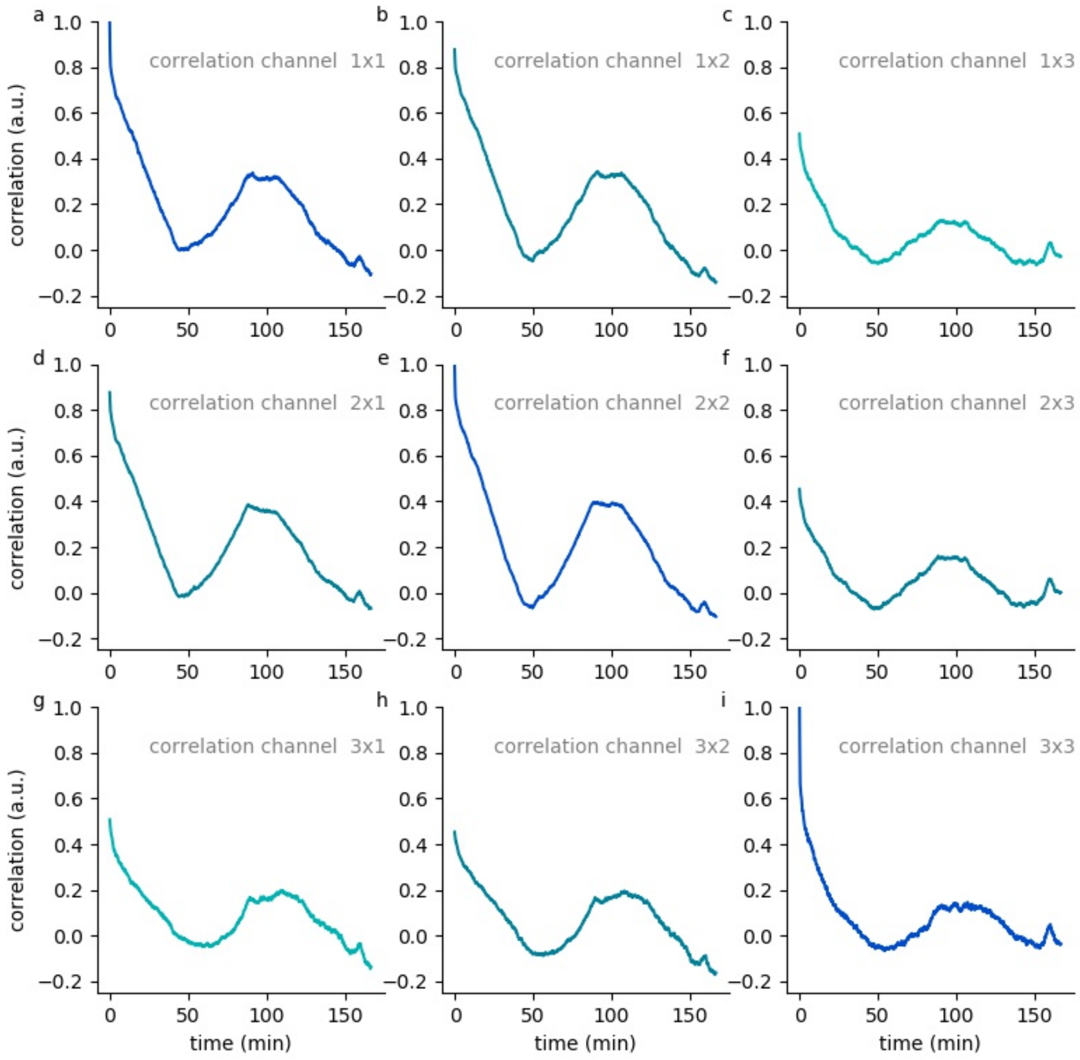

Figure S11: **Hypnodensity cycles.**
Temporal auto- and cross correlations of 5-dimensional hypnodensity probability vectors of sleep stages. Shown are date from subject 56.

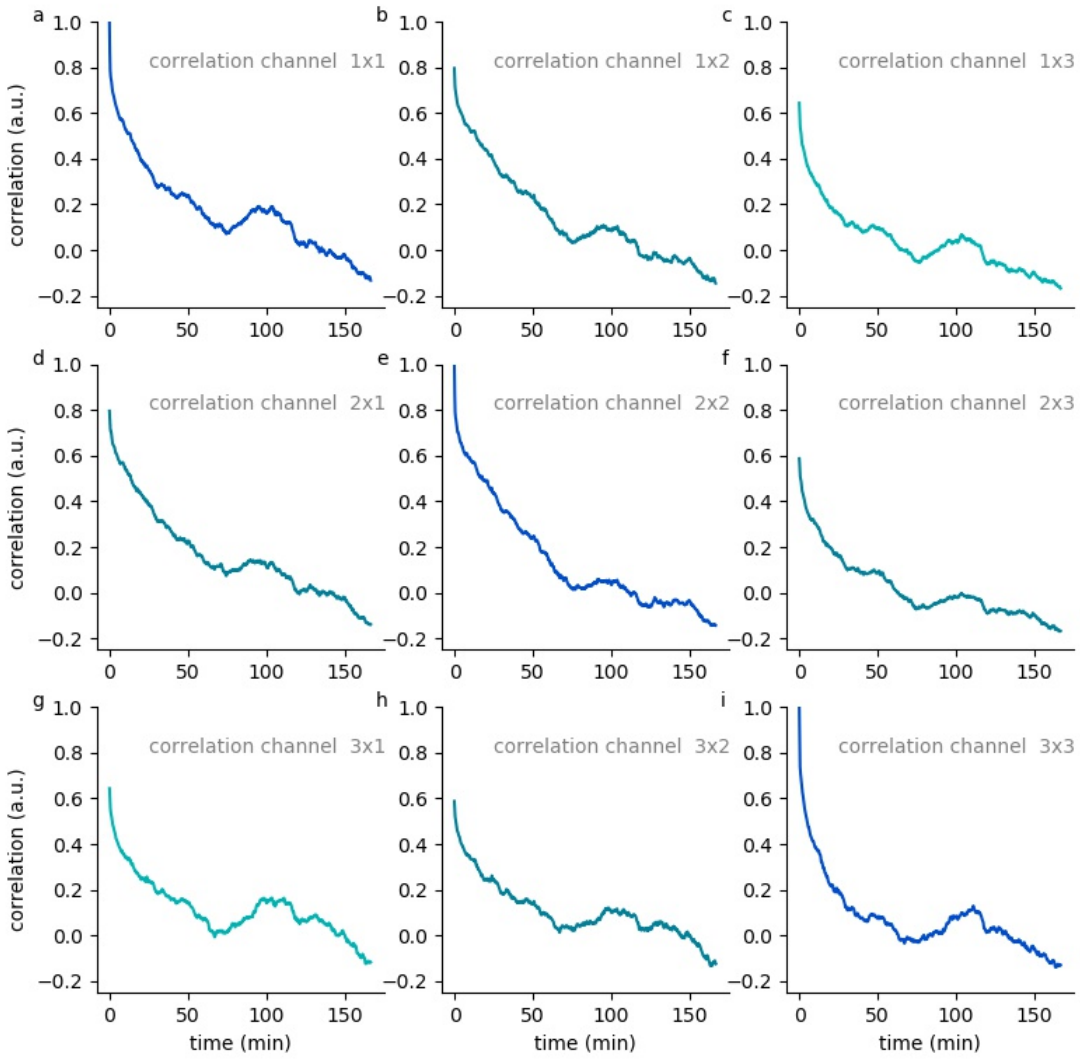

Figure S12: **Hypnodensity cycles.**
Temporal auto- and cross correlations of 5-dimensional hypnodensity probability vectors of sleep stages. Shown are date from subject 57.

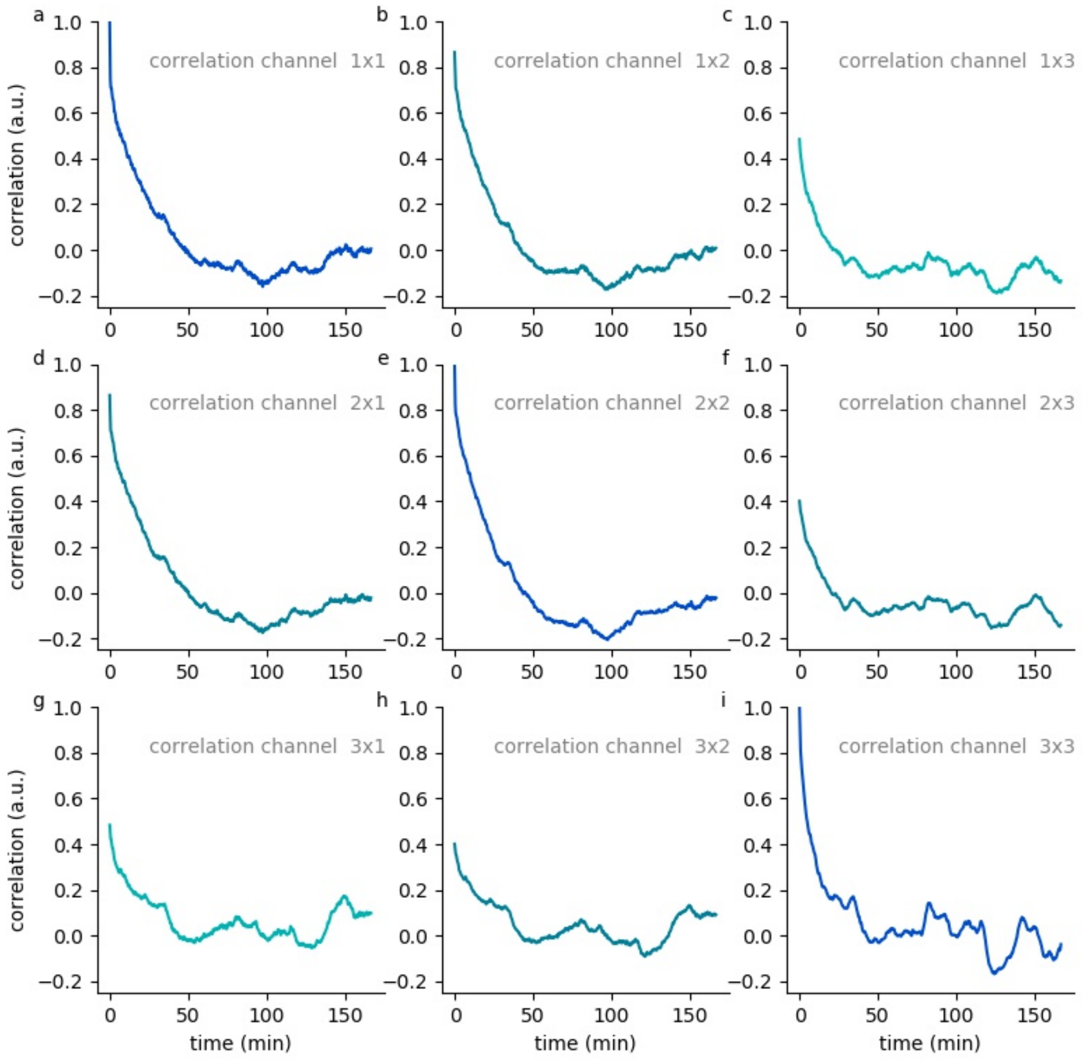

Figure S13: **Hypnodensity cycles.**
Temporal auto- and cross correlations of 5-dimensional hypnodensity probability vectors of sleep stages. Shown are date from subject 58.

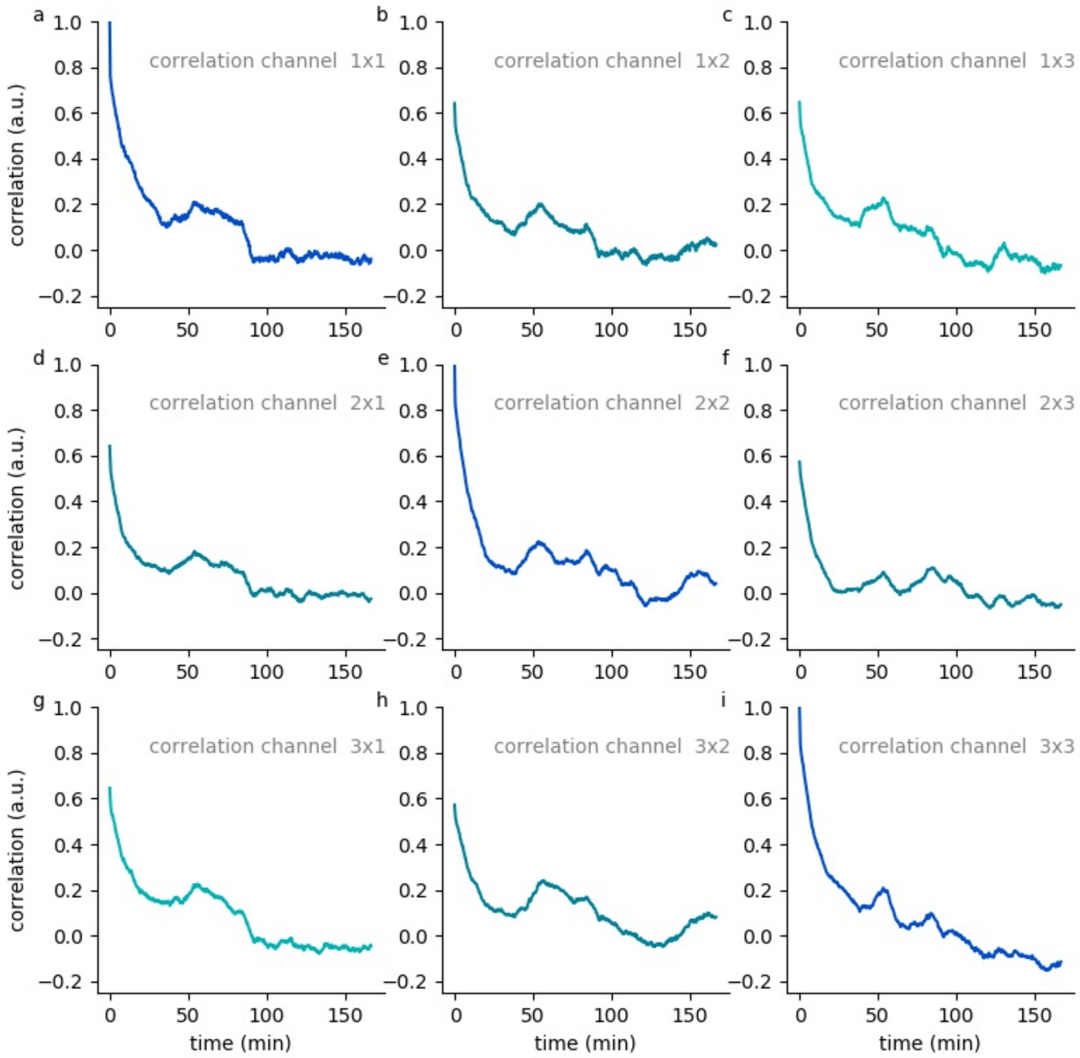

Figure S14: **Hypnodensity cycles.**
Temporal auto- and cross correlations of 5-dimensional hypnodensity probability vectors of sleep stages. Shown are date from subject 60.

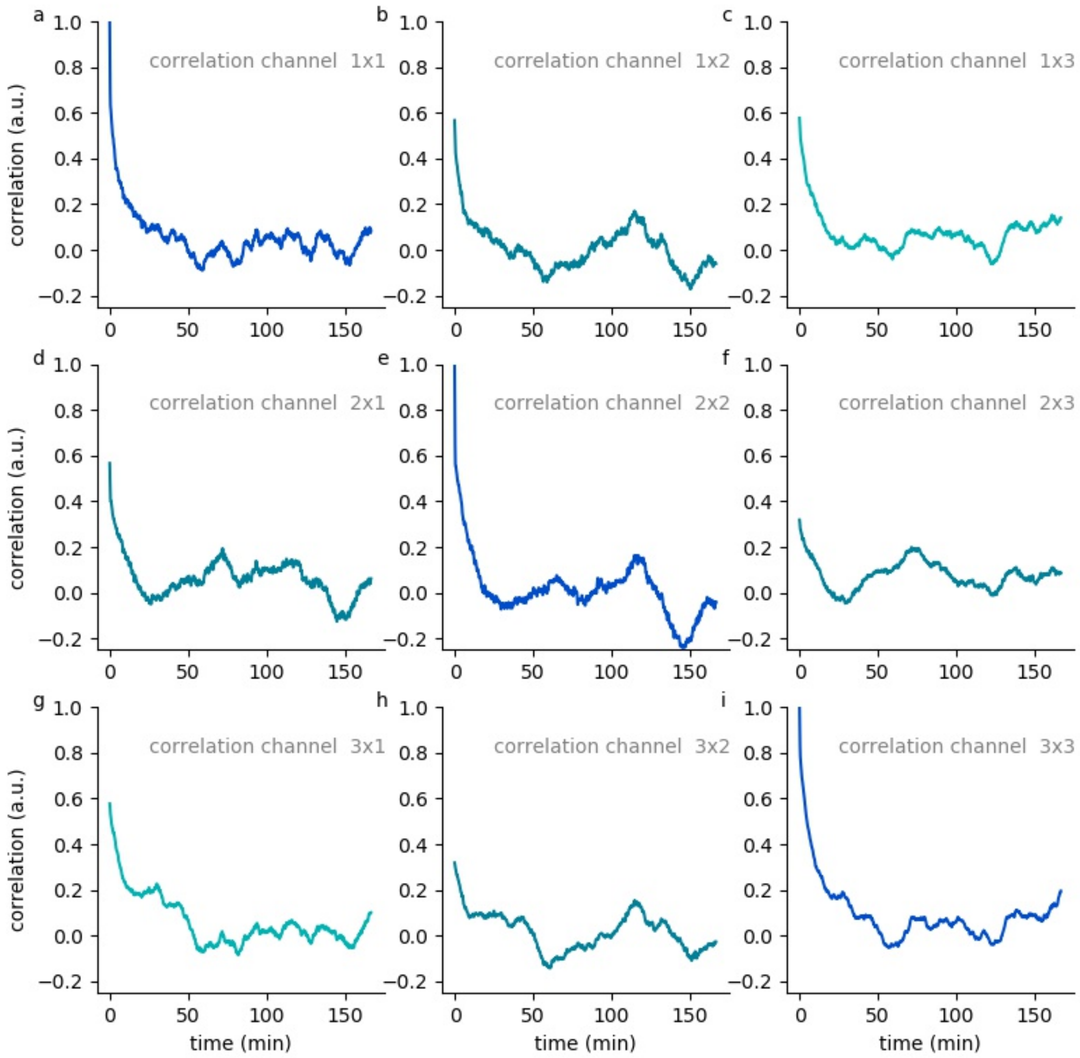

Figure S15: **Hypnodensity cycles.**
Temporal auto- and cross correlations of 5-dimensional hypnodensity probability vectors of sleep stages. Shown are date from subject 61.

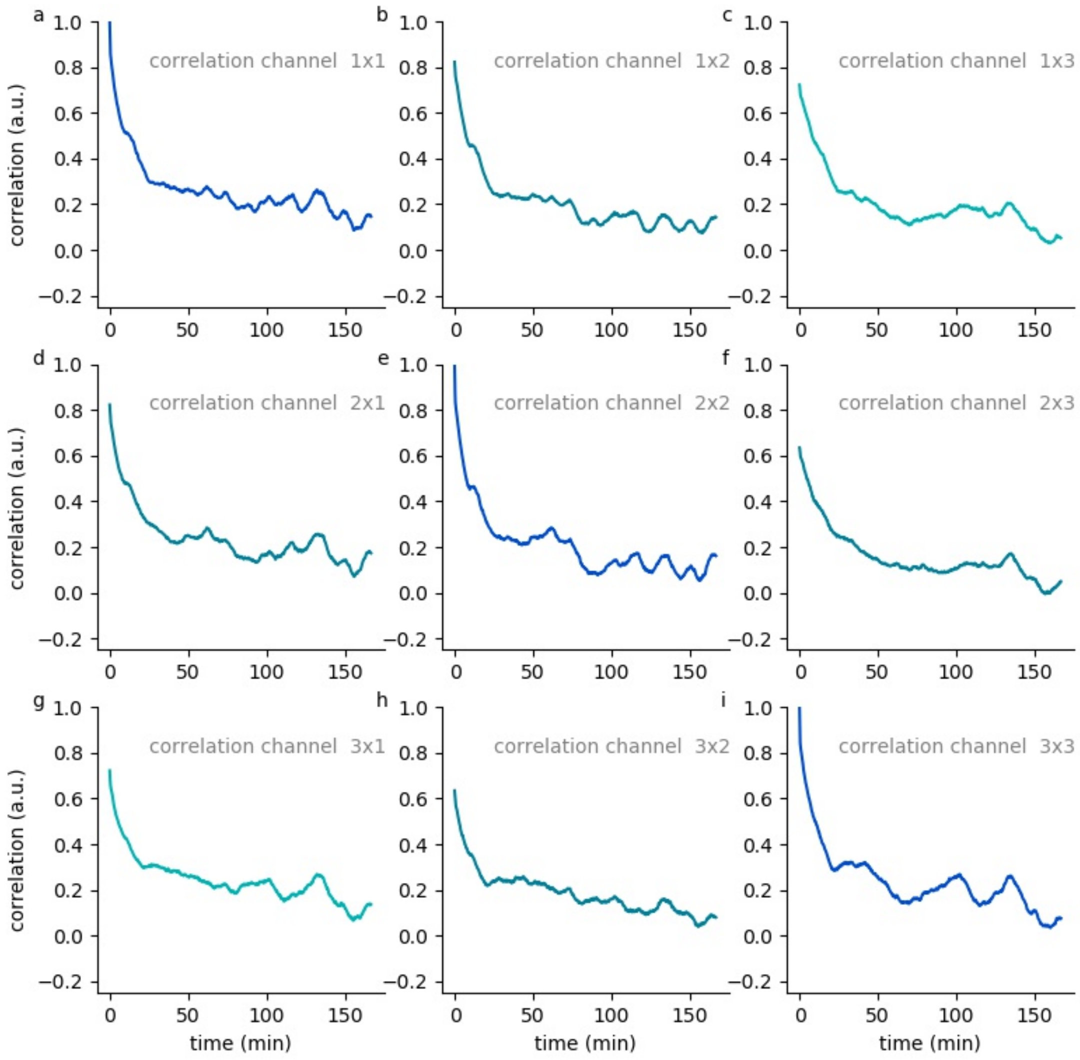

Figure S16: **Hypnodensity cycles.**
Temporal auto- and cross correlations of 5-dimensional hypnodensity probability vectors of sleep stages. Shown are date from subject 62.

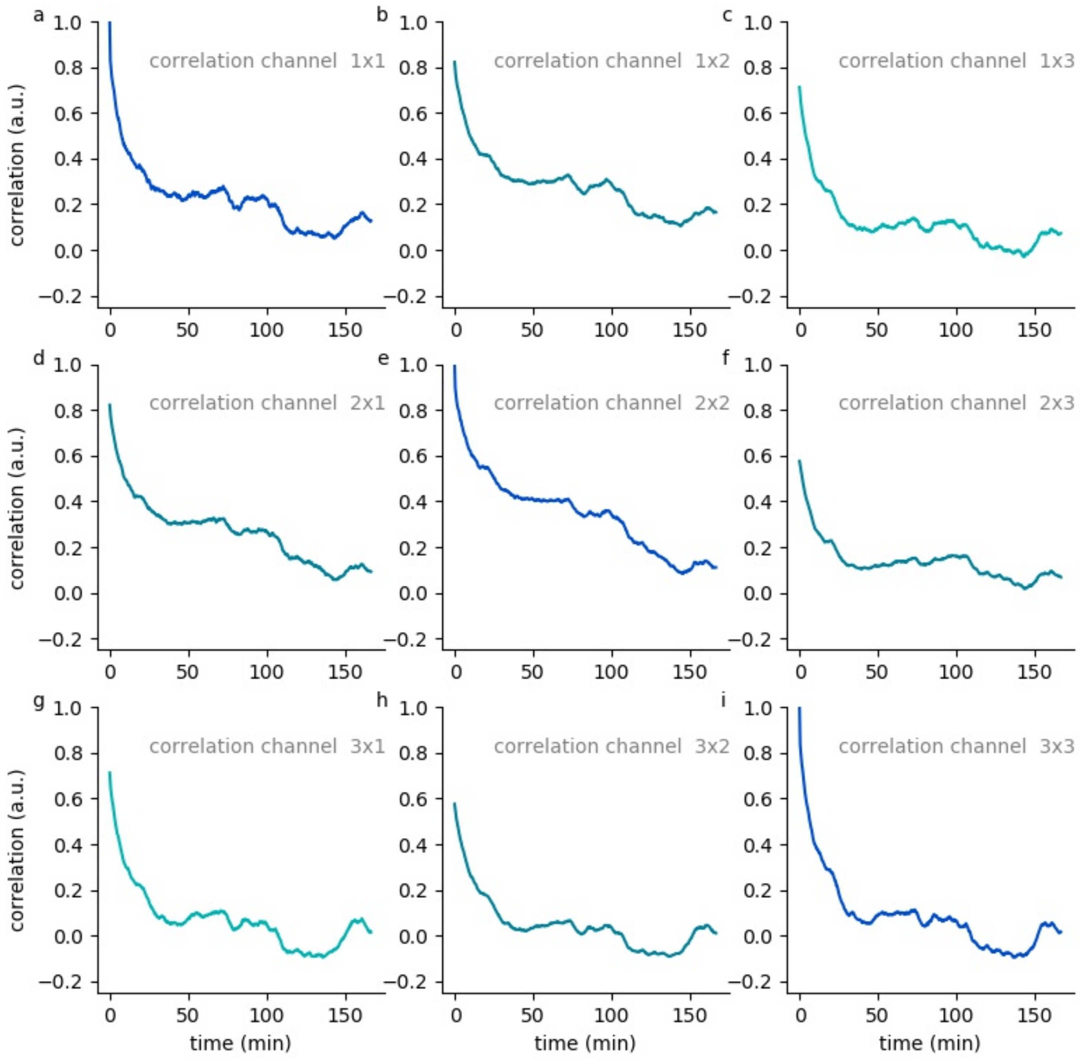

Figure S17: **Hypnodensity cycles.**
Temporal auto- and cross correlations of 5-dimensional hypnodensity probability vectors of sleep stages. Shown are date from subject 63.

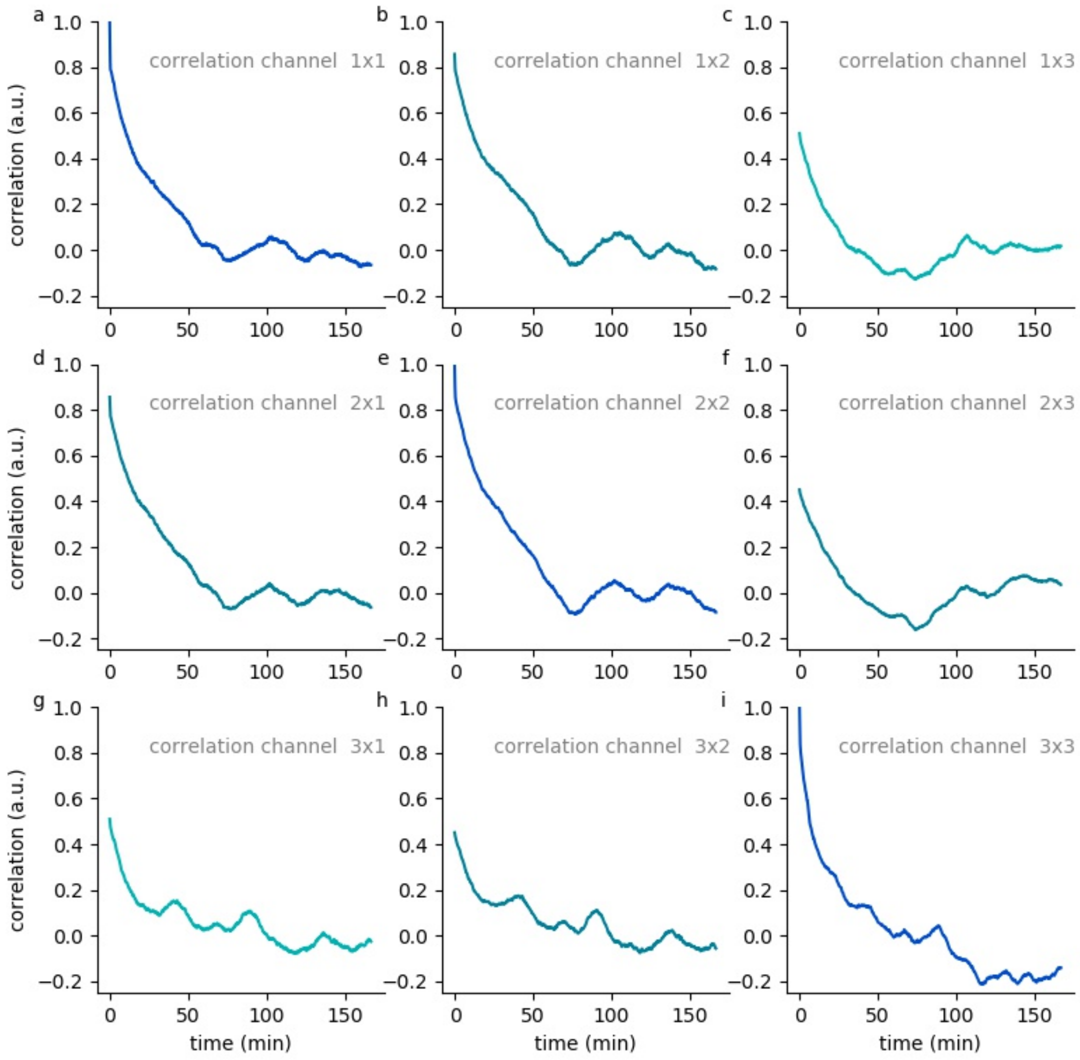

Figure S18: **Hypnodensity cycles.**
Temporal auto- and cross correlations of 5-dimensional hypnodensity probability vectors of sleep stages. Shown are date from subject 64.

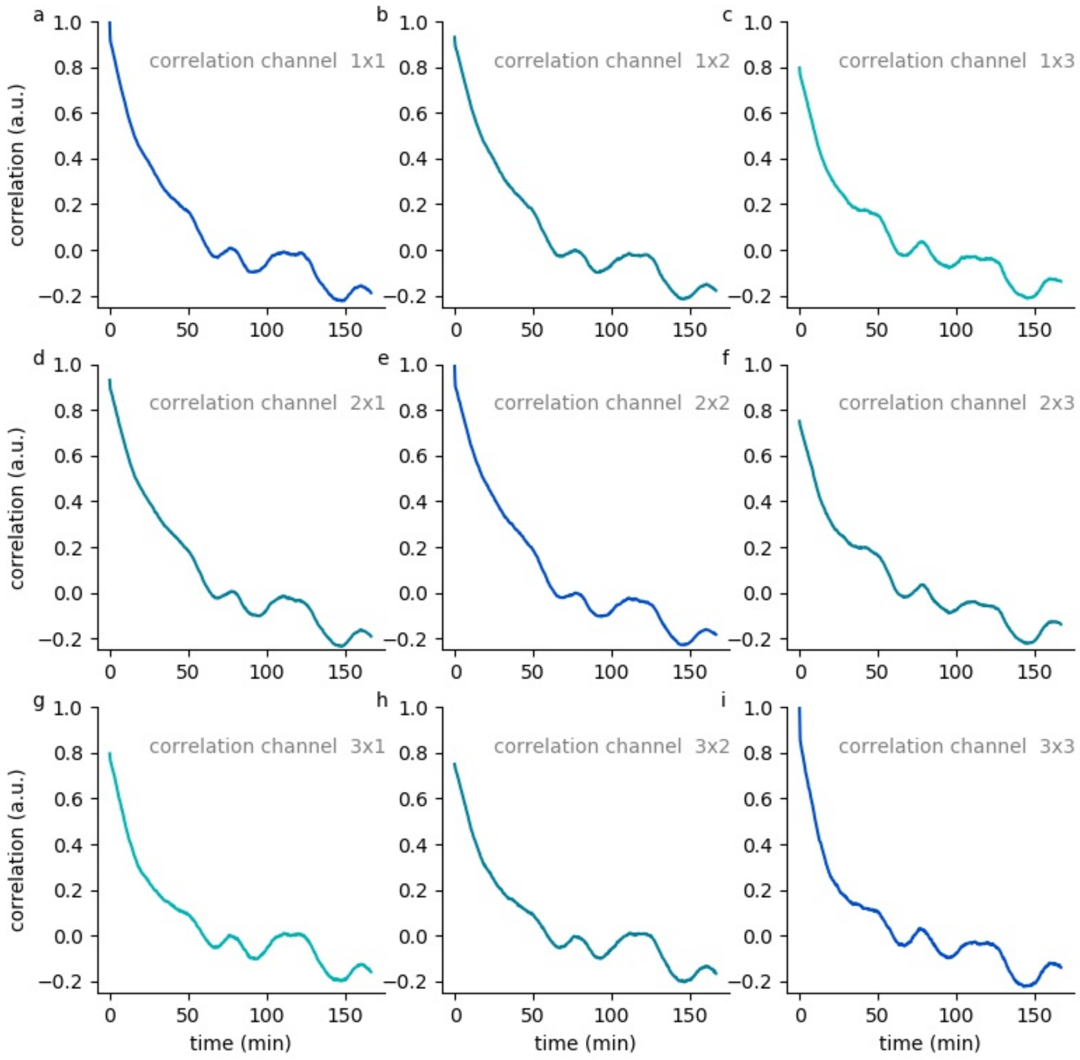

Figure S19: **Hypnodensity cycles.**
Temporal auto- and cross correlations of 5-dimensional hypnodensity probability vectors of sleep stages. Shown are date from subject 65.
